# The protective role of breastfeeding in multiple sclerosis: Latest evidence and practical considerations

**DOI:** 10.3389/fneur.2022.1090133

**Published:** 2023-01-24

**Authors:** Sara Collorone, Srikirti Kodali, Ahmed T. Toosy

**Affiliations:** NMR Research Unit, Department of Neuroinflammation, Queen Square MS Centre, Faculty of Brain Sciences, UCL Queen Square Institute of Neurology, University College London, London, United Kingdom

**Keywords:** multiple sclerosis, breastfeeding, pregnancy, immune system, drug therapy

## Abstract

The immunoprotective role of pregnancy in multiple sclerosis (MS) has been known for decades. Conversely, there has been rich debate on the topic of breastfeeding and disease activity in MS. In clinical practice, women are often offered to restart their disease-modifying drug (DMD) soon after delivery to maintain their relapse risk protection. Limited available information about peri-partum DMD safety can discourage women to choose breastfeeding, despite the World Health Organization's recommendation to breastfeed children for the first 6 months of life exclusively. New evidence is emerging about the protective role of exclusive breastfeeding on relapse rate. Research studies shed light on the hormonal and immunological mechanisms driving the risk of relapses during pregnancy and postpartum. Finally, case reports, real-world data, and clinical trials are increasing our knowledge of the safety of DMDs for the fetus and infant. While some DMDs must be avoided, others may be considered in highly active pregnant or lactating women with MS. This mini-review conveys recent evidence regarding the protective role of exclusive breastfeeding in MS and offers clinicians practical considerations for a patient-tailored approach.

## Introduction

Since the PRISM study in 1998 (1), neurologists have reported that the likelihood of a woman experiencing a multiple sclerosis (MS) relapse was reduced during pregnancy, especially during the third trimester, and again increases in the first 3–6 months postpartum, compared with her pre-pregnancy risk status (1–3).

In this scenario, the effect of breastfeeding on postpartum relapses was unclear: some studies showed potential benefits (4, 5), and others did not (6, 7). Consequently, in clinical practice, women were often offered to restart their disease-modifying drug (DMD) soon after delivery to reduce the chances of having an MS relapse. Limited available evidence about DMD safety meant many women avoided breastfeeding, despite the World Health Organization's recommendation to exclusively breastfeed children for the first 6 months of life (8).

This mini-review discusses recent evidence about a protective role for breastfeeding and provides clinicians with a practical tool for a patient-driven approach in evaluating women with MS planning to breastfeed.

## The biological mechanisms

**Figure 1:**
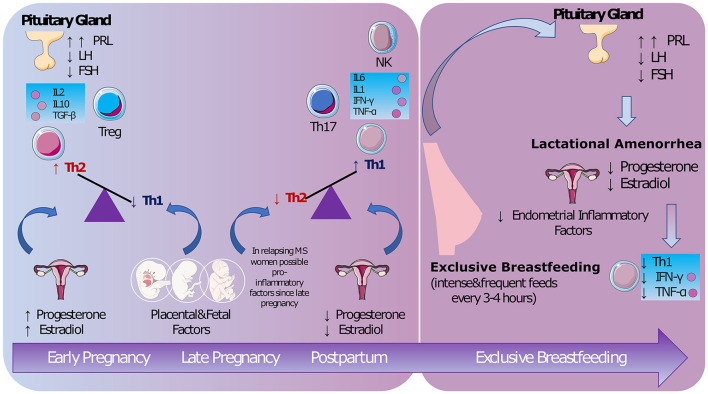
The biological mechanisms. During pregnancy both hormonal and fetal/placental factors maintain a non-inflammatory status. A pro-inflammatory response is restored after delivery [possibly from late pregnancy (5)]. Lactational amenorrhea promoted by exclusive breastfeeding seems to decrease lymphocyte Th1 activity (9). PRL, prolactin; LH, luteinizing hormone; FSH, follicle-stimulating hormone; IL, interleukin; TGF, transforming growth factor; Treg, regulatory T cells; Th, lymphocyte T-helper; NK, natural killer; IFN, interferon; TNF, tumor necrosis factor (Images from Servier Medical Art by Servier https://smart.servier.com/).

To understand the biological mechanisms behind the protective role of exclusive breastfeeding in MS, we consider the hormonal and immunological changes characterizing women's reproductive years, from menstruation to pregnancy and puerperium (Figure 1).

The protective role of pregnancy, which MS shares with other autoimmune conditions, has been known for decades (1).

During pregnancy, there is a shift from a lymphocyte T-helper (Th)-1 [interferon-γ (IFN-γ), tumor necrosis factor-α (TNF-α), interleukin (IL)-2] to a Th-2 (IL-4, IL-10) immune response to protect the fetal-placental unit (10). High levels of estradiol and progesterone suppress lymphocyte Th-1 activity (11), which plays a crucial role in promoting MS inflammatory activity, while placental factors sustain a Th-2 response (10). The effects of estrogen on MS activity have been studied in experimental autoimmune encephalomyelitis (12), cuprizone mice (13), and in clinical trials using estriol (14, 15).

As drop in sex hormones after delivery is associated with increased annualized relapse rate (ARR) postpartum, researchers suggested starting protective treatments from the third trimester or immediately postpartum (16).

However, not all women with MS experience rebound activity after delivery. Other immunological factors may influence the changes in relapse risk in pregnancy and postpartum, such as changes in lymphocyte T-regulatory cells (Th-17) (17) and natural killer cells (18) that occur from late pregnancy. In these studies, however, researchers did not investigate if exclusive breastfeeding impacted the immunological changes.

Researchers started to observe reduced ARR in MS women who exclusively breastfeed compared with non-breastfeeding women (5). A small study showed that MS women having relapses postpartum had increasing levels of IFN-gamma from the third trimester, but this remained low in women who exclusively breastfed (9).

Exclusive and frequent feedings (every 3–4 h) maintain high prolactin and low luteinizing hormone (LH) levels, inducing ovarian suppression (lactational amenorrhea). Since menstrual cycles are involve inflammatory processes that probably serve to prepare the endometrium for embryonic implantation (19), their suppression is could be related to anti-inflammatory effects (20). Prolactin has a controversial role: on the one hand, this hormone has recognized inflammatory activity (21), on the other hand, prolactin may help oligodendrocyte precursors to repair damaged myelin (22).

Whether a predisposition emerges during pregnancy has yet to be defined, and studies that carefully evaluate the role of breastfeeding are still lacking. Furthermore, other factors such as parity, body mass index, and fetal sex may influence the postpartum ARR risk (23).

For instance, fetal sex may influence maternal immune activity during pregnancy. A study found that a male fetus may increase maternal levels of pro-inflammatory cytokines (e.g., IL-12p70, IL-21) (24). In fact, histological studies showed that placentae from male premature babies had greater markers of inflammation than female ones, suggesting a more robust maternal immune response to male fetuses (25). However, other studies could not confirm the association between fetal sex and increased maternal production of inflammatory proteins (26). A recent study by Ross et al. considered multiple factors (23), including fetal sex, body mass index and parity, showing different patterns of inflammatory markers in pregnant women. Their findings highlight the complexity of pregnancy and postpartum regarding immune system maternal adaptation, which is likely to be influenced by multiple factors.

## Toward a protective role for breastfeeding in multiple sclerosis

The PRIMS study (1) was the first large prospective study on MS and pregnancy. The effects of exclusively breastfeeding on relapses were not a specified outcome. However, a subgroup analysis reported no associations between breastfeeding and relapse rate in the first 3 months postpartum.

Since then, there has been controversy about the protective role of breastfeeding on postpartum annualized relapse rate (ARR). Prospective and retrospective studies have followed, and, although authors agree that breastfeeding has no adverse effects, some have reported beneficial effects on ARR (27, 28), whilst others have not (16, 29). One of the main biases was that the early studies included patients who were breastfeeding in general, regardless of its exclusivity and duration. As discussed before, it is thought to be lactational amenorrhea, promoted by exclusive breastfeeding, that seems to suppress inflammatory activity.

In 2009, Langer-Gould et al. (5) were the first to differentiate between exclusive and non-exclusive breastfeeding, finding an ARR significantly higher in women who did not exclusively breastfeed, irrespective of their 2-year pre-pregnancy ARR or treatment history. This observation was in line with evidence for the protective role of lactational amenorrhea (Figure 1). A meta-analysis (30) found women with MS who breastfeed were almost half as likely to experience a postpartum relapse as women who did not. However, significant heterogeneity was present (63%), driven mainly by the variable duration of the postpartum follow-up.

Although two large observational studies nourished the debate (31, 32) on breastfeeding and MS, showing different results (4, 7), recent works have supported the benefits of breastfeeding (33, 34), as summarized by a recent meta-analysis (35). Krysko et al. (35), with an overall estimated heterogeneity of 48%, showed that those who breastfed had 37% lower odds of postpartum relapse than those who did not breastfeed or did not exclusively breastfeed postpartum.

## DMDs and breastfeeding

DMDs target neuroinflammation and are approved for patients with relapsing-remitting MS (RRMS) and active secondary-progressive MS (SPMS) patients (36). Approximately 30% of mothers with MS may relapse within the first 3 months postpartum (1). Effective disease management during this challenging period is essential for the wellbeing of the mother and baby.

Nine classes of DMDs with different mechanisms of action and routes of administration are currently available in MS. For each class, there are limited data available, with some studies showing individual variability (37). However, most DMDs for RRMS are not advised during breastfeeding by manufactures, the Food and Drug Administration (FDA) and European Medicines Agency (EMA) (38, 39).

The drug's molecular weight is probably the most critical factor in determining transfer to breast milk, but oral absorption is also crucial for the child. Poorly absorbed drugs are not likely to enter the child's bloodstream and cause pharmacological effects (40).

### Injectables

Although the FDA and EMA advise caution, first-line modest efficacy injectable treatments for RRMS, such as glatiramer acetate (GA) and interferon beta (IFN-β), are considered safe to use in pregnancy and during lactation (41, 42).

### Oral agents

Oral agents, including modest and high-efficacy treatments, must be avoided during pregnancy and breastfeeding (43, 44). Although there have been case studies in the literature showing a very low Relative Infant Dose (RID) in the breastmilk of lactating mothers for dimethyl fumarate (DMF), larger studies are required to establish its safety (45). Cladribine is a potential choice for MS patients who are planning to conceive because it allows conception 6 months after the last dose (46). The evidence suggests withholding breastfeeding for at least 48 h after a dose (47, 48), but manufacturers recommend a 7-day (Europe) or 10-day (US) abstinence period.

### Monoclonal antibodies

Monoclonal antibodies are highly effective DMDs. Due to their high molecular weight, these drugs do not pass into breast milk in significant amounts. Studies show minimal concentrations and, at least in the short term, no negative impact on breastfed infants. Although the evidence for relative safety is sparse in MS, it is widespread in non-neurological inflammatory diseases (49). Natalizumab, a humanized recombinant anti-α4-integrin antibody, is not recommended in the third trimester (50). In the postpartum, although it is detected in breast milk, its RID is <10%, the recommended safety threshold. As the accumulation of the drug in milk was identified in women receiving multiple infusions (51, 52), longer follow-up is needed to inform definitive recommendations for breastfeeding.

For anti-CD20 therapies, e.g., Ocrelizumab and Ofatumumab, only a few studies of humans and infants with small numbers have been performed, mainly due to the prolonged dose interval (50). Therefore, EMA and FDA recommend high caution for adverse effects in infants if its use is unavoidable in the lactating mother. Nevertheless, different guidelines (53, 54) suggest the resumption of anti-CD20 therapies, if needed, after the first weeks of breastfeeding. Vaccinations, which are contraindicated in breastfeeding women, but needed for anti-CD20 therapies, may be discussed with patients. The first prospective study, SOPRANINO (55), on breastfed infants of mothers on ocrelizumab may establish whether the drug is safe during breastfeeding.

Finally, there is no safety information on anti-CD52 therapy (Alemtuzumab). The EMA suggests stopping breastfeeding 4 months after the last infusion, while the FDA asks to balance the risk of the need of feeding to the infant and the clinical need for infusion to the mother.

## Managing a relapse during lactation

High-dose intravenous or oral methylprednisolone is first-line treatment for acute, disabling MS relapses to hasten recovery. It is typically administered for 3 or 5 days (56).

For the mother, potential side effects of corticosteroid exposure on the baby may include suppression of growth, changes in behavior or appetite, acne, and sleep disorders (57). Particular attention should be paid to mothers suffering from postpartum depression.

A study on the concentrations of methylprednisolone in the breast milk of 16 patients with MS showed that transfer of corticosteroids into breast milk is very low (58). Methylprednisolone levels dropped significantly 4 h after intravenous infusion, and the RID was 0.71% of the weight-adjusted maternal dose, which was >10 times lower than the accepted theoretical RID of 10%. It also found that fully breastfed infants would receive a lower dose than their daily cortisol output. These findings are consistent with previous reports obtained in smaller sample sizes (58). If possible, mothers should wait 2–4 h after infusion to further limit the infant's exposure.

MRI with gadolinium to confirm a relapse is not usually necessary in clinical practice. However, if its administration is considered necessary (e.g., in a differential diagnosis with progressive multifocal leukoencephalopathy), most guidelines state that breastfeeding need not be disrupted after a nursing mother receives a gadolinium-containing contrast medium (59), as only small amounts of this molecule are detectable in the milk.

## Managing DMDs during lactation

Prepartum choices influence decisions about DMDs in the postpartum period. DMD initiation/discontinuation must be carefully evaluated and agreed with the patient before family planning. Clinicians should pay particular attention to natalizumab and fingolimod, whose cessation can cause relapse exacerbation during pregnancy and postpartum.

At present, there are no studies directly comparing exclusive breastfeeding vs. DMDs in terms of efficacy on postpartum ARR.

**Figure 2:**
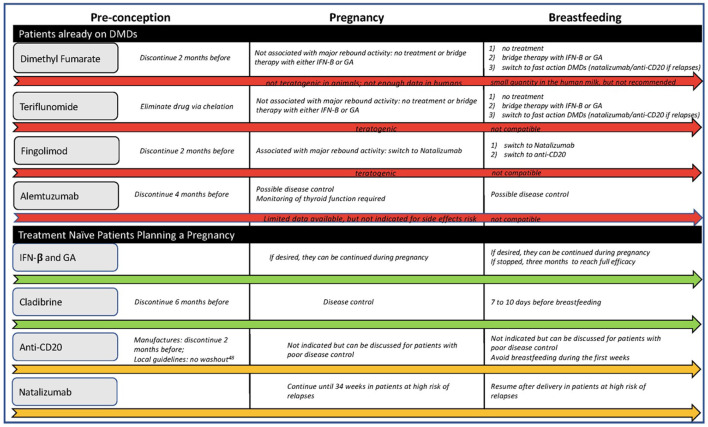
Treatment flowchart. IFN, interferon; GA, glatiramer acetate.

Figure 2 offers a flowchart for possible treatment choices.

### Treatment naive patients

For women planning to conceive at the time of the MS diagnosis, reasonable treatment options include:

- Cladribine, which can be stopped 6 months before conception, offers exceptional control during pregnancy and can be resumed either after breastfeeding or when solids are introduced, allowing seven to 10 days between the last dose and breastfeeding (60) (mothers may want to consider pumping to maintain their milk supply);
- anti-CD20 (ocrelizumab or ofatumumab) because even with exposure around the time of conception, they clear from the maternal system by the time the placental transfer is established and can be resumed during lactation if needed (61);
- GA or IFN, as they can be continued during pregnancy and lactation. However, resuming these drugs in the early postpartum may not affect the risk of relapses (16).

For a patient with high disease activity, defined as frequent relapses with incomplete recovery. natalizumab can be offered, continued up to 28–34 weeks of gestation, and resumed soon after delivery to minimize relapse risk (50, 62).

### Patients already on DMDs

If the patient is not on one of the DMDs mentioned above, a careful plan for treatment cessation should be in place before conception.

Dimethyl fumarate requires a two-month washout period, whilst Teriflunomide needs to be eliminated via chelation. Studies and clinical trials have not associated these two DMDs with rebound activity (43, 62), so it is safe to conclude that patients can resume them after breastfeeding unless pregnancy or postpartum relapses occur. According to the United States label (63), mothers can potentially resume dimethyl fumarate during lactation, but clinicians should monitor infants for potential side effects and weight gain.

Cessation of Sphingosine-1-Phosphate receptor modulators can be associated with rebound disease activity. Authors have also reported fatal outcomes and pregnancy discontinuation caused by severe relapses after fingolimod termination (64). Clinicians should inform patients of childbearing age of these risks. The washout period from fingolimod is at least 2 months, though the availability of ponesimod, which has a shorter half-life, may reduce this time range. Pregnant patients previously on S1P inhibitors can switch to natalizumab and continue for up to 34 weeks of gestation and resume it early after delivery, which, as discussed, can be compatible with breastfeeding. Otherwise, clinicians should carefully monitor the patients during pregnancy and postpartum introducing rapid-action drugs, such as anti-CD20 or natalizumab, if a relapse occurs.

The last infusion of Alemtuzumab should be administered at least 4 months before conception. Lymphocyte depletion should protect women from relapses during pregnancy, but there is no data on breastfeeding, so a second infusion should be postponed until after breastfeeding. Patients should be closely monitored during postpartum and switched to other monoclonal antibodies if there are signs of disease activity before the second infusion.

As discussed before, both FDA and EMA have now dropped the “Category C” classification for pregnancy and breastfeeding from the GA and IFN labels. Consequently, clinicians can consider switching MS patients from potentially teratogenic DMDs to these injectables. However, evidence on the safety of switching during pregnancy and postpartum is very limited. Most existing studies included patients who switched therapy either because of treatment failure (65) or for safety issues (66). Thus, ARR may have influenced the results in the former studies, and both stable and unstable patients were included in the latter studies.

Hence, in pregnant patients on teratogenic DMDs with low risk of rebound activity, clinicians may discuss with the patients the choice of remaining treatment-free during pregnancy and postpartum or using GA and INF as bridging treatment before re-starting the prepartum DMD.

## Current research gaps

### Type of studies

For breastfeeding and MS, evidence only comes from observational studies. Over the years, increasing attention toward women-related issues in MS has stimulated the setup of well-designed prospective studies. However, many studies are still retrospective with the number of relapses based on patients' recall.

### Immortal person-time bias

For many studies, an inclusion criterion for the breastfeeding group is to breastfeed for at least 2 months. Therefore, women experiencing a relapse before this time are automatically assigned to the non-breastfeeding cohort. However, this creates bias (i.e., the immortal person-time bias) by artificially decreasing the number of relapsing patients in the breastfeeding group. Only one study included this bias by defining the inclusion criterion for the breastfeeding group as the intent to breastfeed exclusively for at least 2 months and not actual breastfeeding (34).

### Breastfeeding report and non-exclusively

It is challenging to quantify exclusive breastfeeding in the research setting. In retrospective studies, occasional integration with formula milk may not be correctly recalled. Furthermore, mothers may over report breastfeeding because of social or family pressure. In addition, although Hellwig et al. (34) suggest that benefits are limited to exclusive breastfeeding, no studies have focused so far on partial breastfeeding only. Midwives usually advise exclusively breastfeeding for at least 6 weeks to let the milk supplies stabilize. Afterward, women may decide to integrate with formula milk, occasionally, possibly maintaining ovarian suppression. However, studies usually either group together exclusive and non-exclusive breastfeeding or do not stratify the non-exclusive breastfeeding cohort according to the number of feeds missed in a day. Finally, no studies have investigated breastfeeding along with the hormonal levels to get a further inside on the biological mechanisms behind possible relapse prevention.

### Disease activity

Krysko et al. possibly highlight that the benefits of exclusive breastfeeding are more significant in the presence of high ARR pre-pregnancy (35). However, this evidence is based on few studies, as many authors did not adjust their analysis for prepartum MS activity. In addition, MS treatment is the primary factor preventing women from breastfeeding, potentially biasing the observations.

### Benefits in the long time

Finally, it is not clear how long the benefits of exclusive breastfeeding may last over time. Most of the studies have only accounted for relapses in the first year postpartum. However, two studies extended the observation period and found prolonged beneficial effects on ARR (67, 68). Real-world evidence showed that breastfeeding did not substantially modify the risk of disability over time (69), but prospective studies with a longer observational time are still required.

## Conclusions

The primary biological mechanism driving the protective role of exclusive breastfeeding is ovarian suppression. This suppression is usually maintained until the infant has been weaned consistently to solids (usually around 6–9 months postpartum). Therefore, it is safe to conclude that women with MS can exclusively breastfeed until solids are needed, and this could have a protective effect for at least 1 year (35). More evidence is required for longer durations. Although it has been highlighted (35) that higher prepartum ARRs might be even related to lower postpartum ARRs in exclusively breastfeeding women, further studies must confirm this. Therefore, neurologists should adopt a careful approach for women with high prepartum ARR. Finally, MS women planning pregnancy should be offered multi-disciplinary consultations to decide on their treatment journey. Particular attention should be paid to DMDs that have a relatively high risk of rebound. Then, during pregnancy and breastfeeding, MS women should be carefully monitored for relapses and DMDs should be reinstituted if indicated.

## Author contributions

SC has done the figures included in the manuscript. All authors have contributed in the literature review and in drafting the manuscript. All authors contributed to the article and approved the submitted version.
